# Using Protein Clusters from Whole Proteomes to Construct and Augment a Dendrogram

**DOI:** 10.1155/2013/191586

**Published:** 2013-02-20

**Authors:** Yunyun Zhou, Douglas R. Call, Shira L. Broschat

**Affiliations:** ^1^School of Electrical Engineering and Computer Science, Washington State University, P.O. Box 642752, Pullman, WA 99164-2752, USA; ^2^Paul G. Allen School for Global Animal Health, Washington State University, P.O. Box 642752, Pullman, WA 99164-2752, USA; ^3^Department of Veterinary Microbiology and Pathology, Washington State University, P.O. Box 642752, Pullman, WA 99164-2752, USA

## Abstract

In this paper we present a new ab initio approach for constructing an unrooted dendrogram using protein clusters, an approach that has the potential for estimating relationships among several thousands of species based on their putative proteomes. We employ an open-source software program called *pClust* that was developed for use in metagenomic studies. Sequence alignment is performed by *pClust* using the Smith-Waterman algorithm, which is known to give optimal alignment and, hence, greater accuracy than BLAST-based methods. Protein clusters generated by *pClust* are used to create protein profiles for each species in the dendrogram, these profiles forming a correlation filter library for use with a new taxon. To augment the dendrogram with a new taxon, a protein profile for the taxon is created using BLASTp, and this new taxon is placed into a position within the dendrogram corresponding to the highest correlation with profiles in the correlation filter library. This work was initiated because of our interest in plasmids, and each step is illustrated using proteomes from Gram-negative bacterial plasmids. Proteomes for 527 plasmids were used to generate the dendrogram, and to demonstrate the utility of the insertion algorithm twelve recently sequenced pAKD plasmids were used to augment the dendrogram.

## 1. Introduction

The availability of complete proteomes for hundreds of thousands of species provides an unprecedented opportunity to study genetic relationships among a large number of species. However, the necessary software tools for handling massive amounts of data must first be developed before we can exploit the availability of these proteomes. Currently the tools used for clustering either are restricted in terms of the number of proteomes that can be examined because of the time required to obtain results or else are restricted in terms of their sensitivity. For example, clustering by means of hidden markov models (HMM), multiple sequence alignment, and pairwise sequence alignment by means of the Smith-Waterman alignment algorithm are limited by their time complexity. The Smith-Waterman algorithm, a dynamic programming algorithm, is known to give optimal alignment between two protein sequences for a given similarity matrix [1], but alignment of two sequences of lengths *m* and *n* requires *O*(*mn*) time. On the other hand, heuristic approximate alignment methods, frequently based on BLAST and its variants [2], reduce the computational time required; for example, in practice BLAST effectively reduces the time to *O*(*n*), but this comes at the risk of losing sensitivity to homology detection. In fact, numerous articles—for example, see [3, 4]—have discussed this loss of sensitivity in BLAST-based results compared to those of the Smith-Waterman algorithm. To ensure that a maximum number of homologous sequences are identified, highly sensitive pairwise homology detection is required. Otherwise, the clusters of homologous sequences obtained by means of a given clustering method will not include all possible members and, ultimately, the final results will be less accurate.

In this work we use an alternative sequence comparison algorithm and clustering method called *pClust*. Rather than approximating Smith-Waterman, *pClust* systematically eliminates sequence pairs with little likelihood of having alignments and then only employs the Smith-Waterman algorithm on promising pairs [5]. Clustering is accomplished using a method based on a previously developed approach called shingling [6]. By filtering out unlikely sequences and using the Smith-Waterman algorithm judiciously, *pClust* remains highly sensitive to sequence homology without loss of speed. In an unpublished study of 6,602 proteins from four bacterial proteomes, *pClust* and BLAST results were compared, and BLASTp missed more than 69% of the aligned pairs identified by *pClust*. In a different study, a direct clusters-to-clusters comparison was performed with BLAST results used as the test and *pClust* results used as the benchmark [7]. The results showed that all the BLAST results were included within the *pClust* results but BLAST missed 14% of the clustered pairs obtained with *pClust*. In addition to its sensitivity and speed, *pClust* is readily parallelizable, and to cluster proteins from the proteomes of thousands of species will require high-performance computing platforms and the use of parallel algorithms.

This work was initiated by our interest in plasmids. We wanted a software tool that would allow us to obtain genetic relationships among 527 Gram-negative bacterial plasmids based on their putative proteome sequences. In addition, we wanted an efficient means of adding new plasmids to our initial dendrogram as their proteomes become available. Plasmids are typically circular DNA sequences that can transfer between and replicate within bacteria and that are generally classified as broad- or narrow-host range [8, 9]. Plasmid sequences are described as mosaic because they are composed of DNA arising from many sources [10]. Plasmids serve to shuttle important adaptive traits, such as antibiotic resistance, between organisms [11, 12]. Consequently, understanding the genetic relationships among plasmids is important, for example, in the study of microbial evolution, in medical epidemiology, and in assessing the dissemination of antibiotic resistance genes [13, 14]. There are a number of approaches to examine plasmid relationships. Some researchers focus on the identification of important plasmid backbone genes that are involved in horizontal gene transfer (HGT) or replication within bacterial hosts [15, 16]. Some approaches compare compositional features such as genomic signatures and codon usage [5, 17]. Some researchers use network-based representations to explore genetic relationships among plasmids [5, 18, 19]. In this work we use the whole proteomes of 527 Gram-negative (GN) bacterial plasmids to construct a dendrogram.

**Figure 1:**
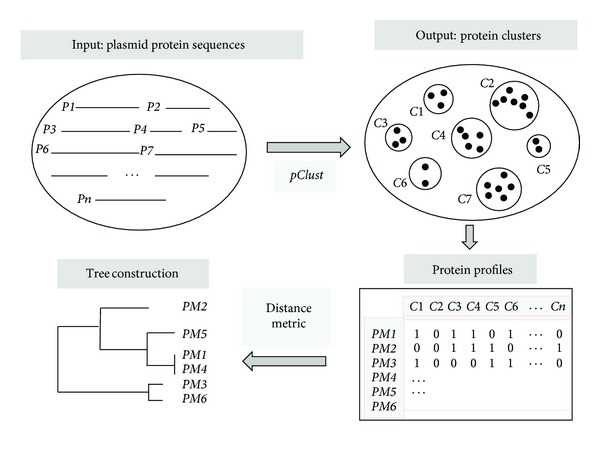
Flowchart for tree construction using *pClust*.

We use protein cluster information from *pClust* to construct our dendrogram and then to predict the relationship of new plasmids within the structure of this tree. A binary profile is created for each species, indicating the presence or absence of a protein in each cluster (Figure 1). The concatenation of all the profiles results in a binary matrix from which a distance matrix is calculated, and neighbor joining is then used to construct a dendrogram. The binary matrix also can be viewed as a library of individual profiles that can serve as correlation filters for a new taxon. A profile for a new taxon can be quickly correlated with the profiles in the library to filter out the profile with the highest correlation coefficient. This correlation coefficient is then evaluated based on known biological information and a decision is made as to whether the taxon should be added to the tree. If it is to be added, its binary profile is added to the binary matrix, a new distance matrix is calculated, and neighbor joining is again used to construct a new dendrogram with the additional taxon. To utilize the algorithm for new plasmids, we focus on sequences from twelve pAKD plasmids that were isolated from Norwegian soil [20]. These plasmids belong to incompatibility groups IncP-1(*β*) and IncP-1(*ε*). A phylogenetic tree constructed using multiple alignment of the relaxase gene *traI* is presented by Sen et al. [20] and serves as a basis of comparison for our augmentation results.

## 2. Materials and Methods

### 2.1. Data Preparation

Zhou et al. [21] presented a virtual hybridization method to construct a dendrogram for 527 GN bacterial plasmids with 50 or more putative coding genes. The same plasmids are used in this study to facilitate comparison. BLASTp with default parameters was used to remove duplicate proteins within plasmid sequences using a similarity score defined by the formula (length of matching sequence)∗(BLAST identity score)/(length of reference protein + length of matching sequence) ≥0.45—that is, proteins with scores ≥0.45 were considered to be duplicates [22]. The maximum score 0.5 is obtained when two proteins are an exact match. Including the matching sequence length in the denominator of the formula insures that a large difference in sequence lengths does not bias the results. After removal of duplicate proteins, more than 97,000 protein sequences remained.

### 2.2. Dendrogram Construction

The flowchart in Figure 1 shows the approach used to construct a dendrogram for the plasmids based on the >97,000 plasmid protein sequences. The protein sequences *P*1, *P*2,…, *Pn* are used as input into the *pClust* program [5], which employs the Smith-Waterman algorithm to perform pairwise comparison of a subset of the sequences. The output from *pClust* is composed of clusters *C*1, *C*2,…, *Cm* of homologous proteins. Protein profiles *PM*1, *PM*2,…, *PM* *n* are then created for all the plasmids from the *pClust* output files. Each profile consists of a binary sequence with 1 indicating the presence of a protein and 0 indicating absence (Figure 1). The *pClust* software was used with default settings in the configuration file except for ExactMatchLen for which a value of 4 was used. A total of 6,618 clusters (defined as having at least two proteins) were identified by *pClust*. The resulting 527 × 6,618 binary matrix was used to construct the dendrogram for two different distance measures. The Jaccard distance metric was originally developed for computation with binary matrices and is given by

(1) $$\begin{array}{c} d_{ij}=\frac{\left(q+r\right)}{\left(p+q+r\right)}, \end{array}$$

where *q* is the number of clusters *C*1, *C*2,…, *Cn* that are 1 for species *i* and 0 for species *j*, *r* is the number of clusters that are 0 for species *i* and 1 for species *j*, and *p* is the number of clusters that are 1 for both species *i* and *j*. We also employ a conventional Euclidean distance metric. For both metrics, a neighbor-joining algorithm was used to obtain the final dendrogram.

### 2.3. Insertion of New Plasmids

As additional plasmid gene sequences become available, we can repeat the procedure described in the previous section to obtain a new dendrogram. The amount of computation and time required to accomplish this task, however, is excessive considering the incremental gain that may be achieved. For example, the original execution time for the 527-plasmid tree was 72 hours on an Intel Xeon CPU E5420 machine with 32 GB of memory. Instead it is preferable to have a means of inserting new plasmids into the existing tree structure as described in this section, where execution of the insertion algorithm takes only a few minutes on a laptop computer.

**Figure 2:**
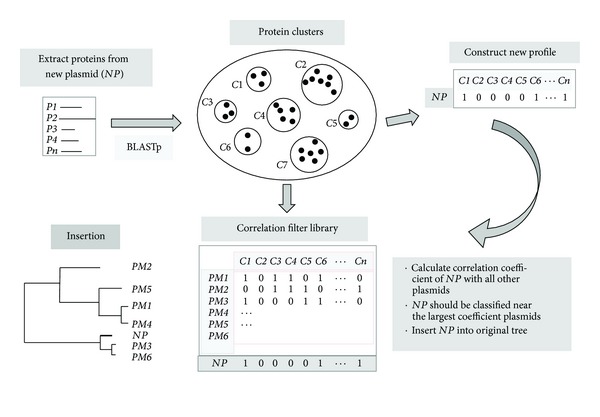
Flowchart for insertion of a new taxon into an existing tree using a correlation filter library.

To insert a new plasmid into an existing dendrogram, proteins *P*1, *P*2,…, *Pn* from a new plasmid are extracted from the plasmid proteome (Figure 2). BLASTp is performed with these proteins against all the proteins in the 6,618 clusters to determine the protein profile for the new plasmid. A protein is considered to be a member of a cluster when its similarity score is >0.2. The similarity score is given by (length of matching sequence)∗(BLAST identity score)/(length of reference protein + length of matching sequence). The cutoff value of 0.2 is consistent with the 40% sequence similarity used as a parameter setting in *pClust.* Correlation filtering is then performed with the correlation filter library consisting of the protein profiles of the original 527 GN bacterial plasmids. The Pearson's product-moment correlation coefficient, whose absolute value is less than or equal to 1, is used to measure the correlation between two profiles [23, 24]. The larger the correlation value, the greater the similarity between two profiles. This value is used to determine whether the plasmid fits into the dendrogram and, if so, where it should be located as explained in the discussion section. When appropriate, the new protein profile is added to the binary matrix, and a tree is constructed from the entire matrix as described in the previous section.

## 3. Results and Discussions

### 3.1. 527-Plasmid Dendrogram

**Figure 3:**
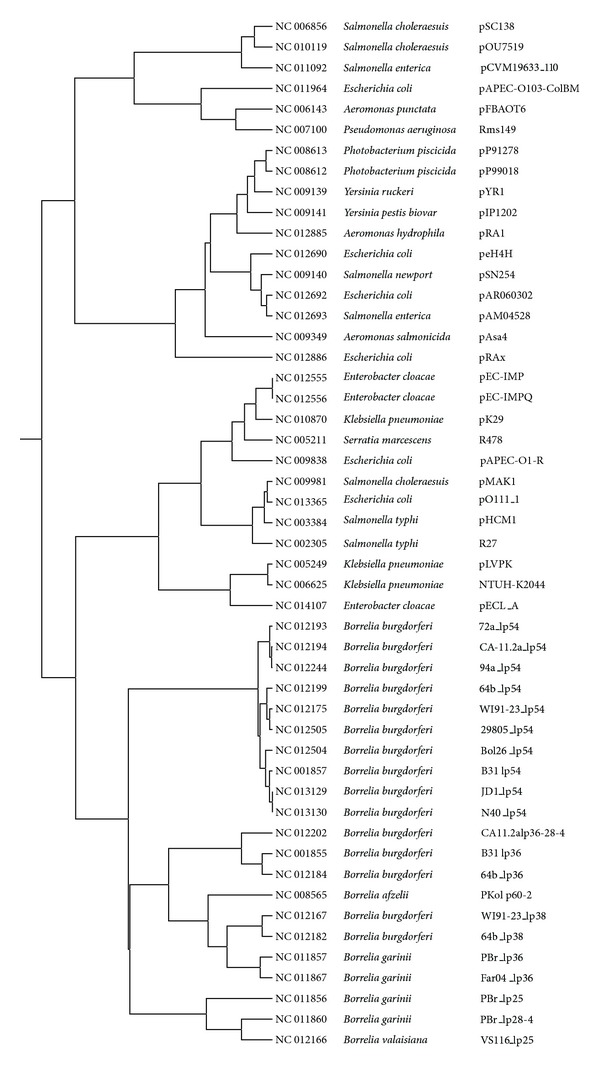
Jaccard distance tree for 50 Gram-negative plasmids.

**Figure 4:**
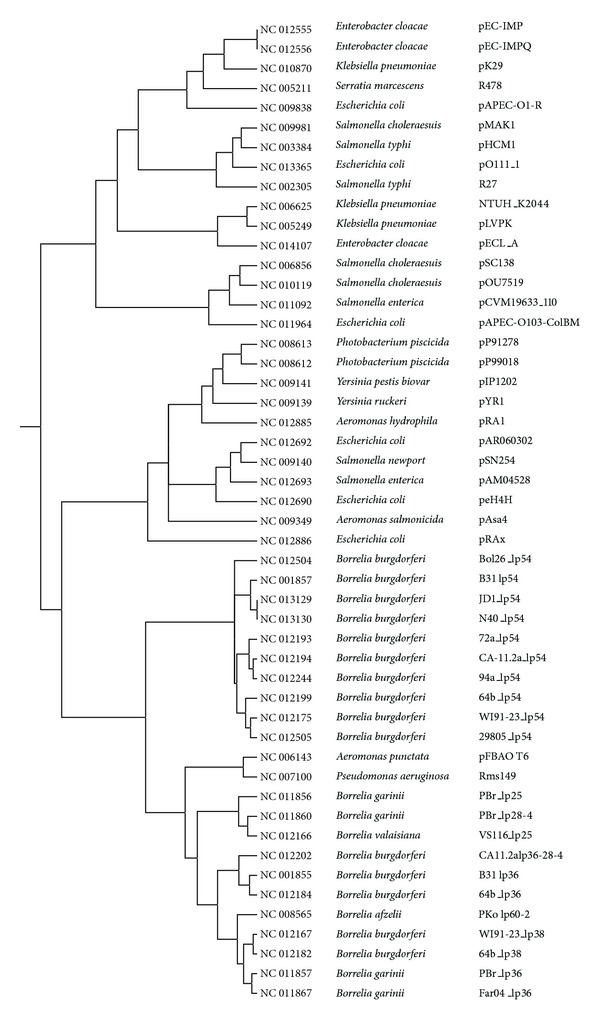
Euclidean distance tree for 50 Gram-negative plasmids.

Following the procedure described above, a dendrogram was constructed for 527 GN bacterial plasmids. Because of its size, it is not shown, but it is available as supplementary information in Newick standard format (.nwk) for both Jaccard and Euclidean distance metrics and can be viewed using MEGA5 [25]. A tree constructed using the Jaccard distance metric for the same subset of 50 plasmids used in [21] is shown in Figure 3, and the Euclidean distance version is shown in Figure 4. These trees are very similar with only a slight difference in the clustering of the *Borrelia* plasmids. The tree constructed using the Euclidean distance metric is closer to the one shown in [21], but the Jaccard tree does a better job of clustering the *Borrelia* plasmids [26, 27]. The Jaccard distance metric is commonly used for a binary matrix. Nevertheless, the results based on Euclidean distance compare favorably with those obtained for a nonbinary intensity matrix using a different approach [21]. It is not clear which distance method gives more accurate results so users should use both matrices and the decision as to which one is more accurate should be determined on the basis of the biology of the system.

### 3.2. Insertion of New Plasmids

**Figure 5:**
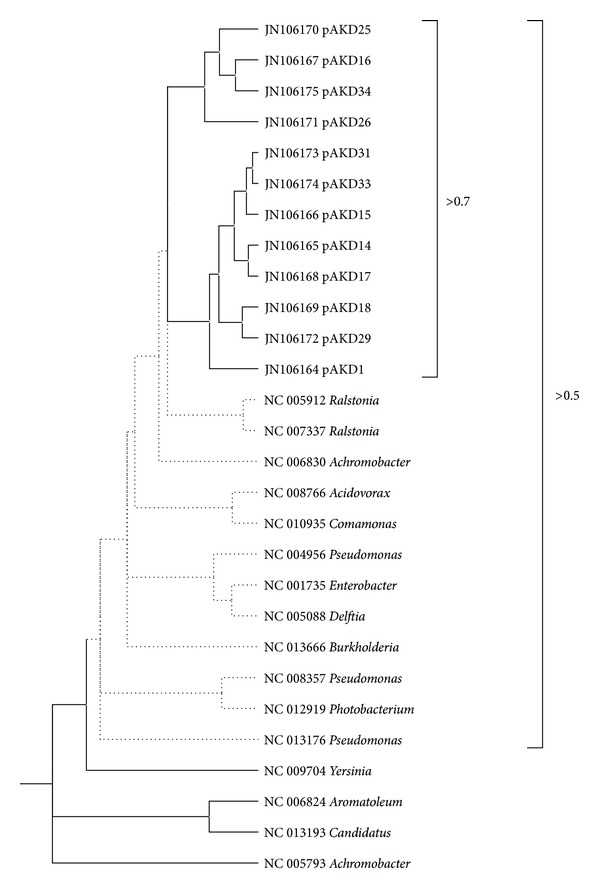
Subtree for 12 pAKD plasmids.

We applied our correlation filter algorithm to twelve new plasmids from the pAKD family [20]. The twelve plasmids cluster together and are most closely grouped with genera typical of other soil bacteria. The correlation coefficient values among the pAKD plasmids were >0.7 and decreased relative to the other plasmids with distance to >0.5 (Figure 5). pAKD plasmids 16, 25, and 34 belong to the IncP-1(*ε*) compatibility group and form a discrete cluster: pAKD plasmids 1, 14, 15, 17, 18, 29, 31, and 33 cluster as the IncP-1(*β*) compatibility group. Although pAKD26 falls into the IncP-1(*ε*) clade, it should be in the IncP-1(*β*) group if compatibility grouping is considered the gold standard for comparison. Nevertheless, the placement is distal from the eight other plasmids in the *β* group, and pAKD26 was actually designated as IncP-1*β*-2 to differentiate it from the other eight plasmids as recently described in [28]. Our results are consistent with [20].

Importantly, the correlation coefficient is used to check the final dendrogram—that is, a new plasmid should be located near the plasmid with which it is most highly correlated. In addition, the correlation coefficient is used to determine whether a plasmid should even be inserted into a dendrogram. In other words, how does the magnitude of the correlation coefficient influence our confidence in the placement of a new plasmid within an existing dendrogram? Several works offer guidelines for the interpretation of a correlation coefficient [29, 30], but all criteria are in some way arbitrary and ultimately interpretation of a correlation coefficient depends on the purpose. In our case, we chose a value of 0.5, but we also require biological evidence—for example, that a plasmid is, in fact, from a GN bacterium.

To further examine the correlation coefficient, we randomly selected 10 Gram-positive bacterial plasmid proteomes from 10 different genera. The correlation coefficients were found to range from 0.112 to 0.234. GP bacterial plasmids do not belong in our GN bacterial plasmid dendrogram, and our minimum correlation value of 0.5 suffices to exclude these unrelated plasmids. While this level of discrimination is easy to identify, we should note that the 527 GN bacterial plasmids considered in this study do not represent the full diversity of GN plasmids. Thus, it is possible to obtain a small correlation coefficient value for a completely new and uncharacterized GN plasmid. If the new plasmid is able to meet an underlying correlation threshold, it can be placed within the dendrogram structure, and by incorporating the new plasmid sequence information into the correlation filter library, we can group future plasmids that may be closely related to it.

While the method of inserting new plasmids into an existing tree is fast and efficient, at some point, generation of a new dendrogram using all proteins from all the taxa will probably be required. We do not know at what point this will occur, but we assume it will be necessary eventually to insure that all possible protein clusters are included. Recall that a cluster must contain at least two proteins to be considered a cluster. Thus, any new plasmid containing a protein that would have formed a cluster with a single discarded protein represents incomplete information in the library. It is probable that the total number of clusters for all Gram-negative plasmids will ultimately be much greater than 6,818.

## 4. Conclusion

In this work we present a new ab initio method for constructing a dendrogram from whole proteomes that begins with output from *pClust*, a software program developed for homology detection for large-scale protein sequence analyses. We develop an efficient approach for insertion of a new species into the dendrogram based on the use of a correlation filter library. This is much more efficient than constructing an entirely new tree which is computationally costly. We illustrate our method by creating a dendrogram for 527 Gram-negative bacterial plasmids and augmenting this dendrogram with twelve pAKD plasmids isolated from Norwegian soil. For purposes of comparison, we also construct a smaller dendrogram consisting of 50 species and use two different distance metrics. The two resulting trees agree well with results shown in [21]. The classification results for the twelve plasmids agree with a phylogenetic tree constructed using multiple sequence alignment of the relaxase gene *traI* presented in [20].

## Supplementary Material

Supplementary File 1: Newick file of Jaccard distance tree for 527 Gram-negative plasmids.
Supplementary File 2: Newick file of Euclidean distance tree for 527 Gram-negative plasmids.
